# Challenges to biobanking in LMICs during COVID-19: time to reconceptualise research ethics guidance for pandemics and public health emergencies?

**DOI:** 10.1136/medethics-2020-106858

**Published:** 2021-05-12

**Authors:** Shenuka Singh, Rosemary Jean Cadigan, Keymanthri Moodley

**Affiliations:** 1 Faculty of Medicine and Health Sciences, Centre for Medical Ethics and Law, Stellenbosch University, Cape Town, South Africa; 2 Social Medicine, University of North Carolina at Chapel Hill School of Medicine, Chapel Hill, North Carolina, USA; 3 Faculty of Medicine and Health Sciences, Centre for Medical Ethics and Law, University of Stellenbosch, Stellenbosch, Western Cape, South Africa

**Keywords:** human tissue, research ethics, ethics, public health ethics

## Abstract

Biobanking can promote valuable health research that may lead to significant societal benefits. However, collecting, storing and sharing human samples and data for research purposes present numerous ethical challenges. These challenges are exacerbated when the biobanking efforts aim to facilitate research on public health emergencies and include the sharing of samples and data between low/middle-income countries (LMICs) and high-income countries (HICs). In this article, we explore ethical challenges for COVID-19 biobanking, offering examples from two past infectious disease outbreaks in LMICs where biobanking activities contributed to the perpetuation of global inequities. We focus on how the ethical imperative to promote the common good during public health emergencies can conflict with protecting the interests of biobank participants. We discuss how conducting biobank research under a waiver of informed consent during public health emergencies is ethically permissible, provided guidance is in place to prevent biopiracy and exploitation of vulnerable communities. We also highlight the need for biobank collaborations between LMICs and HICs to promote capacity building and benefit sharing. Finally, we offer guidance to promote the ethical oversight of biobanks and biobank research during the COVID-19 pandemic or other future public health emergencies.

## Introduction

Biobanking is a public health imperative during the COVID-19 pandemic.^1 2^ Collected samples obviously provide pathology data to improve care, but they also carry the potential to facilitate COVID-19-related research now and in the future. Such an imperative for biobanking has forced us to reconsider human research ethics guidance applied to the various activities of biobanking: collecting, storing and sharing human biological samples and data for research use. We might, understandably, wish to relax some research regulations to better and more quickly respond to the pandemic for public health purposes. But which regulations might be appropriate to relax, and to what extent? In addition, biobanking efforts during past infectious disease outbreaks and epidemics reveal ethical dilemmas and vulnerabilities that particularly affect low/middle-income countries (LMICs).^3–5^ How should biobanking efforts in LMICs balance the urgent need for research during public health emergencies like the COVID-19 pandemic with the ethical complexities of such research efforts, especially given the existing operational challenges that often face biobanking efforts in the LMICs^6^?

This article provides ethics guidance on the operationalisation and oversight of biobanking activities and research in LMICs during public health emergencies. We first discuss how such emergencies, like COVID-19, exacerbate existing tensions between public health ethics and research ethics in biobanking. We then review the ethical challenges of biobanking in LMICs during previous infectious disease outbreaks. From there, we review how COVID-19 has impacted traditional ethical considerations in biobanking, such as informed consent and sharing of samples, and use South Africa as a case study to illustrate how legislative frameworks affecting biobanking might affect biobanking practice in other LMICs during COVID-19. We conclude with practical steps to guide those involved in the ethical oversight of biobanking activities associated with public health emergencies.

## Tensions between traditional research ethics and public health ethics tenets

Ethical considerations in biobanking are typically aligned with those of research ethics, which focus on protecting the rights and interests of participating individuals and communities. Public health ethics, however, espouses ‘pluralistic values’ including population health and the common good.^7^ An ethical paradox arises when a utilitarian emergency social response (a quest for greater common good) unavoidably limits an individual’s right to privacy.^8 9^ Both public health ethics and research ethics invoke principles of solidarity and reciprocity that can be applied similarly in the case of public health emergencies. Both principles emphasise the importance of relationships.

Solidarity asks that individuals recognise a common purpose and willingness to promote the interests of others,^10 11^ whereas reciprocity asks that we balance the risks and benefits of our requests of others and strive to find elements of mutual benefit and equity in these relationships.^12 13^ In the context of a pandemic or other public health emergency, these various ethical principles strongly support global sharing of data and samples^14^ to facilitate diagnostic, preventive and therapeutic interventions for a global population. But they also ask us to consider what may be offered in return—that is, how might the benefits of these actions be allocated?

Benefit sharing gives ‘a portion of advantages/profits derived from the use of human (specimen) resources to the resource providers to achieve justice in exchange’.^15^ More often than not, sharing for LMICs is largely one way: high-income countries (HICs) receive valuable data and samples, whereas LMICs receive little in return. This one-way sharing raises further concerns around ‘compensation for fairness’ as large profits are made using resources from disadvantaged communities.^16^ Benefit-sharing efforts, such as vaccine distribution, that exclude the very communities or countries that provided data and samples for the research can fuel distrust^17^ and suspicion of organisations and individuals involved in biobanking, as well as of the practice itself. Consequently, it is imperative to pay attention to how samples are collected, processed, stored and shared during pandemics and other public health emergencies.

## Biobanking and LMICs in previous infectious disease outbreaks

Here, we explore the ethical challenges of biobanking in LMICs during two recent infectious disease outbreaks, H5N1 and Ebola, and their importance for COVID-19 biobanking practices.

### The H5N1 outbreak: unequal vaccine distribution

In 2007, the Indonesian government refused to share samples of H5N1 virus with the WHO unless they were assured access to the vaccines created from the samples. This decision was spurred by historical inequities in vaccine distribution where people in HICs were more likely to be vaccinated than people in LMICs.^4 5^ In fact, the Indonesian government argued that when LMICs have provided infectious disease data and biosamples to the WHO, pharmaceutical companies in HICs gain access to these samples for free, develop and patent products, and then set unaffordable prices to sell them back to LMICs.^18^ These LMICs tend to have little bargaining power against technologically advanced wealthier countries,^18 19^ which can result in uneven vaccine access.

### The Ebola epidemic: sample acquisition, sharing and biosafety

More than 50 000 samples collected during the recent 2014−16 Ebola epidemic in West Africa were shipped out of the affected countries to unidentified laboratories around the world, primarily in HICs. This shipment was done without state authorisation or proper participant consent,^20–22^ amounting to what some deem biopiracy. Biopiracy could occur when researchers from HICs source samples for academic or commercial gain while failing to fairly compensate communities that provided the samples.^23^ Such acts erode ‘genomic sovereignty’, the ability of LMICs to protect their biogenetic resources.^20^ After the epidemic, an attempt by the WHO to inventory the remaining samples was thwarted by the refusal of some countries—primarily HICs, but also some LMICs— to cooperate in this regard.^21 23^ Some of the concerns related to Ebola samples included the high possibility of infectivity, safety concerns over the handling and storage of such highly infectious samples, and that the samples could be used for bioterrorism.^3 24^ Ultimately, scientists in Sierra Leone, Guinea and Liberia (where the samples were sourced) were unable to access these samples for their research.^25^

### Lessons for COVID-19

These infectious disease outbreaks serve as important examples for biobanking during the COVID-19 pandemic. In fact, similar dynamics are already at play. For example, despite South Africa participating in AstraZeneca COVID-19 vaccine trials, the country has paid double the price per dose compared with European countries.^17 26^ The stockpiling and resulting global maldistribution of COVID-19 vaccines illustrate the persistence of global inequities (WHO 2021), but is also *exacerbating* pre-COVID-19 global inequities in healthcare.^27 28^ These examples also forecast potential ethical issues for COVID-19 biobanking. In terms of sample sharing, early evidence of genetic mutability of SARS-CoV-2 through the appearance of more virulent variant strains was detected by scientists in South Africa.^29^ Yet given worries about the infectivity of COVID-19 and its samples, similar if not greater challenges related to the exclusion of researchers from LMICs from sample sharing may be on the horizon. While global data and sample sharing are imperative during public health emergencies, these examples highlight the need for greater international research ethics guidance and legislation during and after the COVID-19 pandemic to facilitate the sharing of data and the benefits of research with LMICs. Yet COVID-19 has placed unprecedented burdens on hospitals and critical care facilities throughout the world.^30–33^ These burdens, coupled with national lockdowns and other social distancing measures, create distinct ethical challenges for biobanking, which we explore below.

## Biobanking for COVID-19 in LMICs: ethical considerations

Given both its similarities to past infectious disease outbreaks and its unique challenges, governance and oversight for COVID-19 biobanking in LMICs should take into account how the pandemic affects a variety of ethical considerations, which we review below: informed consent, community engagement (CE), sample and data sharing processes, and safe handling of collected samples. Those involved in governance and oversight also need to be aware of the biobanking context, which may affect these ethical considerations. COVID-19 biobanking may occur through new biobanks created as a direct response to COVID-19, as well as through existing biobanks expanding their collection efforts to include the collection of COVID-19 samples or repurposing existing samples for COVID-19 research.^34^

### Informed consent

Informed consent is an ethical cornerstone of research. While volunteerism is important for research participation in general, obtaining informed consent for COVID-19 biobanking research has numerous logistical challenges, some of which may undermine autonomous decision-making. Non-hospitalised patients are instructed to quarantine, making sample collection virtually impossible, and hospitalised patients may be unconscious, heavily sedated, have impaired cognition or otherwise be too sick to engage with the consent process. Similarly, obtaining proxy consent from a patient’s family may be difficult. In South Africa, as in many countries, hospitalised patients with COVID-19 are not permitted visitors, thus limiting opportunities for obtaining proxy consent.^35^ In addition, proxy decision-makers may be understandably anxious and not wishing to engage in a research-informed consent process.^36^ Finally, researchers or others involved in sample collection could be exposed to the virus if proper infection control measures are not in place,^37^ and the availability and access to appropriate personal protective equipment (PPE) have been limited.^38^ When PPE is available, its use, including the use of face masks, can preclude optimal face-to-face informed consent discussions.^36^

Despite logistical difficulties of obtaining informed consent for biobanking, samples are being collected for COVID-19 diagnostic and therapeutic purposes throughout the world. Countries may not have clear regulations that speak to when, if ever, samples collected for these purposes can be repurposed without consent for other research use. For example, in South Africa, health legislation permits collecting samples from hospitalised patients for COVID-19 diagnostic purposes without consent, with provisos, and the Disaster Management Act of 2002 prioritises and makes special allowances for ‘disaster management research.’^39 40^ Yet both pieces of legislation are silent on whether COVID-19 samples can be used for research without consent. Furthermore, while biobanks likely have clear procedures for when samples may be repurposed without consent, data from a recent international survey of biobankers found that many reported that local Research Ethics Committees (RECs)/Institutional Review Boards (IRBs) were more liberal in their permission to waive consent for biobanking of COVID-19 biospecimens than was their usual practice.^34^ If samples are to be collected and stored for COVID-19-related research from hospitalised patients under a waiver of consent, patients who are not severely ill can be informed when they are admitted to the hospital and allowed an opportunity to ask questions and possibly opt out, and posters informing patients and loved ones about the biobanking efforts and purpose may be displayed throughout the hospital.

Collecting and storing samples for research from COVID-19-positive patients under a waiver of consent will have public health benefits and must be weighed against respecting individual patient autonomy. Biobankers should develop a plan to deidentify or anonymise collected samples and data prior to sharing to mitigate any possible risks to individuals.^36^ Waived consent for sample storage during pandemics is arguably justifiable based on necessity provided that future research is COVID-related (or, perhaps, related to other infectious disease outbreaks), in the public interest and approved by an REC/IRB. In addition, existing collections of stored biosamples could be repurposed for research during a pandemic without the need for reconsent provided that studies using such biosamples are appropriately designed and that study benefits are equitably distributed.^41^ The waiver of reconsent should be consistent with the requirements of country-specific legislative frameworks on consent for research purposes. However, mechanisms must be in place to prevent biopiracy or exploitation of vulnerable participants via material transfer agreements (MTAs), data transfer agreements (DTAs) and authentic collaborative relationships in LMICs. In addition, protections must be in place to prevent the use of samples from countries with looser or fewer regulations on repurposing samples, which may lead to the exploitation of those in LMICs.

### Community engagement

CE contributes to procedural justice to ensure fairness in how significant decisions are made in health research.^42^ Engagement activities are important to conduct when members of marginalised communities are expected to participate in the research or when there are social and economic disparities between those conducting the research and those participating. Either of these situations may be the case for biobanking efforts involving LMICs. Biobanking CE efforts are essential for developing public trust in biobanking^42^ as it involves communication about the purpose of the biobank, building relationships and collaborations,^43^ and ensuring communities have a voice in biobanking efforts.^44^ CE is particularly important if informed consent for biobanking or repurposing of samples will not be sought (eg, an REC/IRB waiver of consent). However, engaging with communities in LMICs during COVID-19 is challenging and traditional CE processes may not be possible. Virtual processes may also prove difficult as community members may not have access to electronic platforms or may not have data for use with mobile phones. This, however, should not be a reason to abandon engagement with community members from LMICs, and legislative bodies or RECs should make the integration of CE a priority.^45^

### Sample and data sharing

Coordinated, global sharing of samples and data during a pandemic is critical to expedite research, and biobanks are key in this endeavour. However, prior research indicates that underutilisation of biobank resources is a common problem for biobanks and a source of ethical concern for biobankers.^46 47^ Global sharing of samples and data should be a priority, not only during the pandemic but even when the COVID-19 outbreak has waned. There are opportunities to counter traditional challenges in underutilisation of biosamples that inevitably impact the sustainability of biobanks through coordinated and planned efforts for sample and data sharing.^48^

Individuals involved in biobank governance, such as biobankers, RECs/IRBs and researchers, should be cognisant of the ethical complexities surrounding sample and data sharing. The potential for relaxed research regulations during a pandemic, such as waiving informed consent and decreasing community engagement, increases the imperative for biobanks to strengthen the conditions under which samples and data are accessed and shared. It is not uncommon for samples and data to initially be shared with one group, like academic researchers, only for a third party (such as for-profit companies that fund the research) to gain secondary access.^49^ One possible ethical approach to increase data sharing between institutions could be through the use of secure online data management systems to facilitate collaboration rather than transfer. Thus, data sharing with third parties could occur without the need to transfer proprietary rights to these entities.^49^ Proper governance of biobanks must involve clear protocols detailing the process of deidentification or anonymisation of the samples and data prior to sharing during a pandemic. This is particularly important given the potential for stigmatisation. During COVID-19, various groups have been stigmatised during the pandemic, although the particular groups and the extent of the stigmatisation vary from culture to culture.^50 51^ LMICs face particular risk from the prospect of stigmatisation during a pandemic as the sharing of resources is already ethically fraught, with LMICs often on the losing end. When sharing samples, unambiguous MTAs with clear specifications on how individuals’ and communities’ rights and interests can be protected are imperative. When sharing specimens and data from individuals in LMICs, MTAs should also outline how benefits from any therapeutics or vaccines produced by the research will be shared with the LMICs.

### Safe handling of COVID-19 biosamples

At a biobank operational level, COVID-19 biosamples must be handled safely given their potential infectivity.^52^ Some of the possible adverse consequences related to the processing and storage of COVID-19 biosamples could include accidental spillage of collected samples, risk of exposure, cross-contamination and spread of infection among staff. Thus, reviewing safety measures around the handling of stored coronavirus-related specimens and isolated strains, optimal decontamination processes for the laboratory setting and the availability of PPE for staff is critical^24 38 53^ Researchers or biobanking staff involved in specimen annotation need to ensure that the integrity of the collected samples is optimally maintained, and there should be ongoing skills development to ensure staff competency in dealing with COVID-19 biosamples so that stored biosamples can be used to advance scientific goals.^54^

Biobanks should also consider biosecurity measures when dealing with pathogens. ‘Dual use research of concern’ is scientific research that is intended to benefit society but could cause societal harm if misapplied.^24 55–57^ COVID-19-related research using potentially contagious samples highlights these concerns. The appropriate or beneficial use of research could be to learn more about coronaviruses and treatment regimens, but at the same time such pathogens could also be put to dangerous use such as the creation of bioweapons for bioterrorism.^58 59^ If samples collected during a pandemic could represent a priority target in bioweapon design, vigilance and proper governance in controlling access to stored samples are essential. Such vigilance, however, should not be used as a reason to restrict scientists and researchers in LMICs from having access to data and samples. Legislation at the country level, as well as international collaboration, is required to ensure proper governance and safe sharing of dangerous pathogens (including the need for proper MTAs in the export of samples).^60 61^

## Ongoing ethics review

Given the possible competing tensions of public health ethics and research ethics during public health emergencies, those involved in biobank oversight, such as RECs/IRBs, need to consider innovative, flexible ways to simultaneously protect research participants, promote public health interests and support scientific advancement. Such oversight should include the review of international research collaboration agreements, specifically those that involve partnerships between LMICs and HICs. Such reviews could include questions on whether the research conforms to data sharing, benefit sharing and capacity-building activities between LMICs and HICs.^62^

As new information on COVID-19 is constantly emerging, risk–benefit assessments may need to be reviewed on an ongoing basis in both LMICs and HICs. This effort may require additional training, resources and capacity development of RECs/IRBs and others in LMICs to better respond to the changing research landscape during pandemics, or other public health emergencies. There should be clear collaborative efforts and communication networks between those involved in the oversight of biobanks in LMICs and HICs to ensure adequate support and consistency for decision-making.

In box 1, we offer questions to promote the ethical oversight of biobanks and biobank research related to the current pandemic or future public health emergencies. These questions provide guidance on the review of such related research. This is not an exhaustive list of questions and should be supplemented by other local and context-specific ethics queries. However, this list can serve as the basis for review templates during these pandemics or other public health emergencies. Such templates could also assist in not only standardising the process but also providing additional support for RECs/IRBs.

Box 1 **Questions to promote ethical oversight of biobanks and biobank research during the COVID-19 pandemic or other public health emergencies**

**Respecting research participants and promoting societal benefit**

- Is obtaining informed consent practicable, and would doing so significantly slow research progress?
- Has a risk–benefit assessment been done to determine the appropriateness of relaxing human research subjects’ protections to facilitate the proposed research? For example, are the risks of not obtaining informed consent outweighed by the societal benefits that are likely to accrue from the proposed research? And would local communities likely see any of these benefits?
- Is there equitable participant involvement so as to ensure a fair distribution of possible benefits and burdens of the research, including across LMICs and HICs?
- If using a waiver of consent, are notifications in place so that participants may be made aware of the biobanking research?
- What community engagement activities are planned?
- What country-specific legislation permits collecting, storing and sharing samples for research use without informed consent? What, if any, additional mechanisms are in place to protect the privacy of research participants, such as anonymising samples and data?
- What oversight and monitoring processes are in place for approved research during the public health emergency?

**Sample and data sharing**

- Are there clear plans for sample and data sharing to accelerate research?
- Do research collaborations between LMICs and HICs involve adequate benefit sharing and capacity building?
- Can data be shared via a secure online data management system to facilitate collaboration rather than proprietary transfer?
- What mechanisms are in place, such as MTAs and DTAs, to ensure that resources are not shared with unauthorised third parties?
- What existing legislations, if any, guide sample and data sharing, and do these legislations make allowances for public health emergencies?

**Safe handling of infectious disease biosamples**

- How will biosamples be safely collected from research participants, especially in contexts where PPE resources may be scarce?
- What measures are in place to ensure a safe working environment for researchers and laboratory staff?
- What biosecurity measures are in place?

## Conclusion

The current COVID-19 pandemic provides an opportunity to reflect on ethical considerations in biobanking during public health emergencies, including respect for research participants, promoting the common good, solidarity, reciprocity and benefit sharing. This requires a review of research ethics guidance and regulatory requirements regarding sample collection, storage and sharing for research so that we are better prepared for the next pandemic or public health emergency. Despite the ethical challenges which we have discussed, there is nevertheless an urgent need to optimise sample sharing to accelerate a research platform that could ultimately benefit all affected by public health emergencies. Similarly, any therapeutic or preventive interventions that result from data and/or sample sharing must be fairly distributed, ensuring equitable access to all. Future research should investigate the ways that existing regulations have been relaxed during the pandemic, such as implementation of waivers of consent, and their resulting effects. For example, to what extent will these changes promote research activities? And to what extent have they affected public trust in biobanking? The latter will be particularly important to consider in ongoing discussions of whether and how research ethics guidance for biobanking during pandemics and public health emergencies ought to be reconceptualised.

## Data Availability

There are no data in this work.
